# Equipping family physician trainees as teachers: a qualitative evaluation of a twelve-week module on teaching and learning

**DOI:** 10.1186/1472-6920-14-228

**Published:** 2014-10-22

**Authors:** Marietjie R de Villiers, Francois J Cilliers, Francois Coetzee, Nicoline Herman, Martie van Heusden, Klaus B von Pressentin

**Affiliations:** Family Medicine and Primary Care, Faculty of Medicine and Health Sciences, Stellenbosch University, Stellenbosch, South Africa; Education Development Unit, Faculty of Health Sciences, University of Cape Town, Cape Town, South Africa; Centre for Health Professions Education, Faculty of Medicine and Health Sciences, Stellenbosch University, Stellenbosch, South Africa; Ukwanda Rural Clinical School, Stellenbosch University, Stellenbosch, South Africa; Centre for Teaching and Learning, Stellenbosch University, Stellenbosch, South Africa; Faculty of Medicine and Health Sciences, Stellenbosch University, Stellenbosch, South Africa

**Keywords:** Family physician roles, Capacity-building, Teaching-skills training

## Abstract

**Background:**

There is a dire need to expand the capacity of institutions in Africa to educate health care professionals. Family physicians, as skilled all-rounders at district level, are potentially well placed to contribute to an extended training platform in this context. To play this role, they need to both have an understanding of their specialist role that incorporates teaching and be equipped for their role as trainers of current and future health workers and specialists. A teaching and learning capacity-building module was introduced into a new master’s programme in family medicine at Stellenbosch University, South Africa. We report on the influence of this module on graduates after the first six years.

**Methods:**

A qualitative study was undertaken, interviewing thirteen graduates of the programme. Thematic analysis of data was done by a team comprising tutors and graduates of the programme and an independent researcher. Ethical clearance was obtained.

**Results:**

The module influenced knowledge, skills and attitudes of respondents. Perceptions and evidence of changes in behaviour, changes in practice beyond the individual respondent and benefits to students and patients were apparent. Factors underlying these changes included the role of context and the role of personal factors. Contextual factors included clinical workload and opportunity pressure i.e., the pressure and responsibility to undertake teaching. Personal factors comprised self-confidence, modified attitudes and perceptions towards the roles of a family physician and towards learning and teaching, in addition to the acquisition of knowledge and skills in teaching and learning. The interaction between opportunity pressure and self-confidence influenced the application of what was learned about teaching.

**Conclusions:**

A module on teaching and learning influenced graduates’ perceptions of, and self-reported behaviour relating to, teaching as practicing family physicians. This has important implications for educating family physicians in and for Africa and indirectly on expanding capacity to educate health care professionals in Africa.

## Background

Family medicine offers various benefits as a specialty in the African context. These include the availability of a skilled generalist at the district hospital, mentoring of team-based care in the community, a strong leadership role in the district health system, and developing holistic practice of medicine [1]. Contextual differences between Africa and other parts of the world have prompted initiatives to define the role of family medicine in sub-Saharan Africa. An international consensus process defined the roles of the African family physician as care provider, consultant, capacity-builder, supervisor, manager and collaborator [2, 3]. This places an important focus on the family physician’s capacity building role as the lead clinician in the district hospital.

There is also a renewed focus on training, recruitment and retention of human resources for health in Africa [4]. Amongst others, an increased output of medical doctors via the increased use of decentralised clinical training is advocated. Family physicians’ capacity building role can include facilitating clinical training away from the tertiary/teaching/academic hospital setting (parallel to decentralised quality of care improvement initiatives).

Postgraduate studies in medicine prepare students to play advanced roles in their fields. Typically, this focuses on the utilisation of advanced disciplinary knowledge and skills. However, it is increasingly being recognised that as students graduate from advanced studies, they play leadership roles not only in practicing at an advanced level, but also advancing knowledge in the field and preparing the next generation of practitioners and leaders. It is therefore evident that capacity-building skills training programmes, relevant to the African context, need to be developed and evidence of their effects be sought.

A common format of capacity-building skills training programmes for medical specialties, including family medicine, is that of teaching-skills programmes for residents [5–7]. A systematic review of formats, content and effect of existing programmes for family medicine residents identified 362 studies [7]. Although a wide variety of formats for these programmes exist, the content usually included effective clinical teaching skills, leadership skills, and evaluation and feedback skills. The programmes generally improved teaching behaviours, but further studies are needed to guide the development of context specific effective programmes [7].

To improve the preparation of future family physicians for their teaching role, a decision was taken during the formulation of a new master’s programme in Family Medicine at Stellenbosch University in South Africa in 2003 to include a module on teaching and learning in family medicine (TLFM). (Medical doctors in South Africa may enrol in a Masters of Medicine (MMed) programme after completion of two years internship and one year community service, to qualify in any of the medical specialties, the equivalent of medical residencies in North America.) The overarching aim of the module is to prepare the family physician to be an effective teacher of family medicine in her/his own decentralised setting.

The four year Masters in Family Medicine programme consists of theoretical modules, practical training under supervision and a research project. The TLFM module is presented in the third year of the programme. The educational outcomes for students in this module are as follows:

At the end of this module the learner should be able to:

1. Describe the role of the family physician as both teacher and learner
2. Describe relevant principles of adult education and learning theory
3. Determine learning needs and plan educational activities
4. Use educational technology effectively
5. Deliver a high quality educational presentation
6. Facilitate small group learning
7. Elicit course evaluation and feedback from participants or students
8. Describe the principles of student assessment

The module is presented in two parts, namely a day-long contact session and a 12 week period of guided self-study, facilitated using an online learning management system. The contact session focuses on teaching skills such as presentation and small group skills. The online module is organized in six blocks of two weeks starting with a block interrogating the overall role of the family physician in the African context (Table 1).

**Table 1:** **TLFM module course outline**

Weeks	Content	Practical tasks	% grades
**1–2**	Role of family physician as teacher	Discussion 1	5%
**3–4**	How do people learn	Discussion 2	10%
**5–6**	Planning a teaching activity	Discussion 3	10%
**7–8**	Being an effective teacher	Discussion 4	10%
**9–10**	How to evaluate your teaching	Discussion 5	5%
**11–12**	Perform and evaluate teaching activity	Complete teaching assignment	60%

The students’ self-study is guided by an online study guide which outlines learning opportunities. These include readings, structured asynchronous online discussions with peers and tutors, as well as the posting of reflective summaries. Students are assessed on frequency of participation and quality of discussion postings. The module culminates in an assignment reporting on a teaching intervention designed and delivered during the module. In addition, students are assessed on their presentation skills when presenting their research dissertation in the final exit examination for the Master’s degree.

After presenting the module for six years, it was considered timeous to explore the influence of the module on graduates. In addition, little was found in the literature about similar interventions in an African setting. This article reports on graduates’ perceptions of their role as family physician, attitudes towards teaching, and participation in and confidence with teaching activities.

## Methods

An interpretive and exploratory strategy was adopted in this qualitative research design. The research group consisted of the TLFM module designers and tutors from 2004–2009, (the first six years of implementation) two graduates who completed the module in 2009 and an independent researcher not involved in the presentation of the module.

In-depth interviews were conducted between October 2010 and March 2011 with 13 graduates (2004–2009) of the Master’s in Family Medicine programme, exploring the influence the module has had on them. For pragmatic reasons and to allow for face to face interviews, all respondents were drawn from the province where the researchers were situated (Western Cape province, South Africa). Following an initial written invitation to participate, graduates were followed-up telephonically to confirm participation and schedule interviews.

All interviews were conducted by the independent researcher in the preferred language (English or Afrikaans) of the respondent. The interviewer was fully bilingual. Trigger questions were used to explore the respondents’ perceptions and views about the influence of the TLFM module on their teaching practice. Ethical clearance was obtained from Stellenbosch University’s institutional research committee (reference number N10/07/238). Written informed consent was obtained from the participants and the analysis of the interviews was done after the anonymization of the transcripts.

Interviews were audio-recorded and transcribed in full. All co-workers read and coded the interviews individually to identify first level items that were considered important. Thematic analysis was driven and refined by group discussions to develop themes. *ATLAS.ti* was used to facilitate the analysis. Data saturation was identified by cross-referencing quotes from all the interviews to the themes. There was strong consensus about a number of overarching themes emerging from the data. Quotes in Afrikaans were translated for the purposes of reporting.

## Results

### Respondents and their context

**Figure 1:**
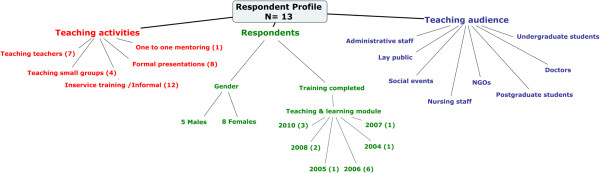
**Profile of respondents.**

Figure 1 summarises the respondent profile including their occupation, teaching activities and teaching audiences. The average age of respondents was 39.5 years (range 34–61). Four respondents worked in family physician posts in the public sector.

### Themes

**Figure 2:**
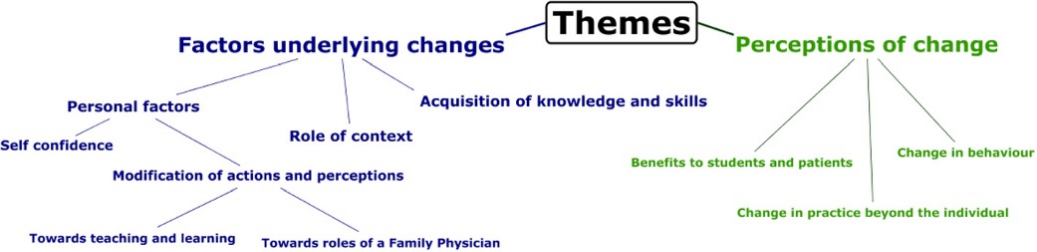
**Themes identified.**

It was evident that themes emerging from the data could be related to the Kirkpatrick framework as adapted by Barr, Freeth, Hammick, Koppel and Reeves [8] and Cilliers and Herman [9]. Our focus was in the first instance perceptions and evidence of change offered by respondents (addressing Kirkpatrick levels 3, 4a, and 4b) (Table 2). We were also interested in the factors underlying these changes, which emerged as the role of context and the role of personal factors. Context was seen as a medley of factors that enable or constrain to varying degrees. The role of personal factors addresses Kirkpatrick levels 1, 2a and 2b. Level 1 results are not reported here. Figure 2 provides an illustration of the themes.

**Table 2:** **Adapted Kirkpatrick’s framework**

Level 1 –	Participants’ reactions
Level 2a –	Modification of attitudes and perceptions
Level 2b –	Acquisition of knowledge and skills
Level 3 –	Change in behaviour
Level 4a –	Change in organisational practice
Level 4b –	Benefits to patients/clients/students

Evidence of changes in behaviour and perceived benefits to various groups resulting from the module will be presented first (Section A). Secondly, the issue of factors potentially underlying these changes will be examined in Section B. Several quotes are cross-referenced to more than one theme. All quotations are numbered (a) numerically at the beginning to allow for cross-referencing; (b) with a respondent number at the end (i.e. R1 for respondent one).

## A perceptions and evidence of change

### A1 Change in behaviour

Respondents described various ways in which their teaching behaviour had changed as a result of the TLFM module (cf. also Quotes #8, #21, #22). Respondents reported actively planning for teaching (cf. also Quote #14); doing presentations differently; and incorporating new teaching techniques such as small group work and the one-minute-preceptor into their teaching repertoire. Some also indicated that they now make time for teaching which they did not do before (cf. Quote #8).

#1 I have learned for the first time about the concept that ‘to teach people doesn’t mean that they are learning’. If you only speak, you very soon lose your ‘audience’. I try to get the audience involved in the conversations, I try to get different opinions on a question while discussing it, and I summarise when I have dealt with a section. I can see that the people do better in the post-test than in the pre-test. (R1)

#2 Group work [was particularly good], where we got participation from students, presentation by the students rather than you teaching them, everybody participated, especially when you gave them specific instructions of role play. Role play was very effective. (R2)

Respondents reported now also taking the trouble to involve the students through needs assessment, good planning, and student feedback on their teaching (cf. Quote #5). Respondents also reported developing and implementing student and preceptor feedback on teaching.

#3 I am newly appointed in my post. Last year I developed a questionnaire for students to evaluate the doctors and a similar one for doctors to evaluate each other. (R3)

Respondents were also able to adapt their teaching to participants’ needs and prior learning (cf. also Quote #9) and adapt what they had learned to suit a variety of circumstances e.g., working with semi-literate health promoters.

#4 The ‘health promoters’ do not really have a medical background … [the skills that I learned in the module help me] definitely. … You work with illiterate people. Therefore the presentation has to change and you have to use more visual aspects and pictures and case scenarios. (R10)

#5 I think one feels more confident…we’ve learned that to teach you’ve got to assess the need… . (R11)

### A2 Change in practice beyond the individual

Although this constituted a smaller theme, a few of the respondents elaborated on examples of how the module had influenced the practice of others. Respondents described how they not only assisted others with their teaching but also deliberately involved others in teaching and in the process taught them how to teach.

#6 …the clinical nurse practitioners, sometimes I assist them with their PowerPoint presentations, but I try to ask them to hone their own presentations, by putting topics together. I only listen to them, I try to moderate. (R11)

One respondent developed flow charts for teaching chronic diseases, which are now used across a health district. Another respondent used the tools acquired in the module to successfully motivate for more staff.

#7 I drew up a management plan for chronic diseases as I realised there was nothing in place. The nurses get training, the doctors get training, but the basic guidelines were still necessary. (R3)

### A3 Perceived benefits to students, patients, community and self

Respondents highlighted examples of how they perceived the module to have benefited their students (cf. Quote #1). They felt that their own self-confidence and motivation for teaching had a positive and empowering effect on their learners.

#8 Previously I used to think of teaching more as a duty. …the students would come… I would have to take them with me on a ward round and show them patients and they would stand there and I wouldn’t know how much they were taking in or how interested they are. Whereas once I started involving them more in the patient or giving them a bit more things to present or think about, I could see how much they were taking in and I could see the level of interest increase. So it became more rewarding…, because you could see there was more interaction. … [I feel motivated by it] and I enjoy it, because… you can see that those who are learning have gotten something out of it. (R2)

#9 [The teaching and learning module] helps me make it more interesting for the staff to learn about something,. The Hospice sisters present their patients and we discuss them and I first try and evaluate what they think is a solution to the problem so then it’s more than just one person teaching the other one, assimilating. It’s more that they discover for themselves what the possibilities are for treatment for their patients. (R2)

Pre- and post-testing provided feedback to learners on their progress in the particular subject matter (cf. Quote #1). Nursing staff were reported to be eager and enthusiastic to learn as appropriate knowledge empowered them to provide better clinical care to their patients. As mentioned earlier, it was felt that there were benefits to patients by way of the new guidelines on disease management and improved clinical knowledge and skills as a result of the teaching.

#10 It is very difficult to measure impact just because I am responsible for the training. There are many other roleplayers and variables. …[tuberculosis] (TB) is better controlled in our district compared to other districts. The sad thing is that nurses work in TB clinics … but cannot answer basic questions about TB. …they are very keen to receive training as was shown in the post test and the type of questions we answer telephonically. The quality of referrals to the hospital has improved in the last two to three years. I think our training has contributed to this improvement. (R1)

There were instances where respondents reported organising training and talks to address organisational issues, a “meta-level” utilisation of insights from the module. Respondents reported organising teaching for colleagues to create a stimulating environment so as to retain staff and decrease turnover. Teaching skills were also utilised in talks to the broader community to raise awareness about the hospital.

#11 I spoke to the gynaecologist in charge of training at the local hospital as I wanted to know how we could get involved. Now we do maternity ward rounds bi-monthly in the mornings. The nursing staff knows that some doctors have not done this recently and that they need to refresh their skills before they could be of value in the wards. The nurses are very keen to train the doctors attending the prenatal clinics. (R3)

#12 I have addressed many groups, for example the local chamber of commerce, the municipality and other similar groups. I have also spoken to church groups and the community. I feel at ease and I actually enjoy … [informing the rural community about the role of the district hospital]. (R9)

Respondents reported feeling less stressed about teaching and also reported the accrual of personal and not just professional benefits.

#13 I suddenly gave more presentations and as a result I was asked to be master of ceremonies at a wedding reception.. It was clear to others that I have gained confidence to speak in public and although it was a minor role initially, it means a lot to me today. I was always confident, but not to get up in an audience and make a contribution. (R1)

## B Factors underlying these changes

In analysing the data it became clear that the context in which respondents function had significant enabling or constraining influences in terms of applying their teaching and learning skills. Their own self-confidence, attitudes and perceptions, as well as knowledge and skills also influenced the degree to which they implemented what they had learnt in the module.

### B1 The role of context

Different contexts offered varying levels of opportunity and support for teaching and learning. Respondents’ job description was an important indication of their sphere of influence in terms of applying the TLFM module knowledge and skills. Of the 13 respondents, four were appointed in family physician posts in the public sector, and one as medical superintendent (Figure 1), where their responsibilities included the teaching and training of students and staff. These respondents were more comprehensively involved in teaching and learning activities. Despite most of the other respondents not having teaching and learning as part of their core job description, most of them indicated enthusiasm for (cf. Quotes #7, #13), and actively seeking out, opportunities to teach.

#14 This module meant so much to me because as a family physician, part of my responsibility in the current post is to do some teaching, limited teaching to undergraduates, doctors from [names university] faculty of health science, and also the residents in family medicine… . So it empowers me to be able to assess their training needs, and where I need to focus and plan my teaching in such a manner that it gives a desired outcome. (R11)

The biggest constraining influence on teaching and learning was the lack of time, amidst a heavy workload of clinical and (for some) managerial duties. Respondents nonetheless displayed a positive approach towards this in terms of making time for teaching.

#15 Obviously in a busy community health centre you don’t get a chance to apply, or to try out different methods… because you have this huge patient load as well. But, you could experiment every now and then and try out something that you had learnt. (R4)

#16 If you want to do teaching and learning there must be protected time and the facility is responsible to create that time and to protect it. The problem is that our facilities are always short staffed and in terms of our work, people do not attend, because if they are not in the unit, there is no one to do the work. (R10)

Logistical challenges such as lack of administrative support and suitable venues were also mentioned as constraints.

#17 The problem is that doctors who have to present these things never really have the necessary administrative support to get started. (R10)

### B2 The role of personal factors

#### B2.1 Self-confidence

All respondents indicated that they felt empowered by the TLFM module to teach using knowledge and skills gained during the module. The module gave respondents the self-confidence to seek and take opportunities to teach (cf. Quote #5). They now enjoyed teaching whereas before, some were hesitant to be involved in teaching due to lack of self-confidence (cf. Quotes #5, #22). They reported feeling able and even confident to adapt their knowledge and skills to novel situations (cf. Quotes #4, #5). A particularly strong theme was the self-confidence they developed to do presentations in varying formats. Some respondents not only used these skills in their professional lives but also socially with some becoming successful public speakers (cf. Quote #12, #13). There was some evidence of how self-confidence in teaching developed. Incremental successes played a role. Respondents reported trying something, with some success, which built self-confidence to be more involved in teaching and try again at another opportunity, with another success which built more confidence (cf. Quotes #13, #22). The role of the planning and execution of a teaching activity as summative assessment for the TLFM module, as well as assessment on presentation skills during the final examination for the course were also emphasized.

#18 I am naturally a shy person who would prefer not to speak in front of a crowd. I gained quite a bit of confidence in doing that, in doing the PowerPoint [at the contact session] and in actually presenting it. (R6)

Knowledge also played a role in attaining self-confidence. A number of respondents indicated that knowledge attained in the module, by giving them an approach to a teaching situation, gave them more confidence (cf. also Quote #27). It was also evident that the nature and manner of feedback respondents received during the module helped empower them.

#19 It increased my confidence because I didn’t realise there were so many different ways to teach, that you could have the patients being presented by the students and then from there go on to teaching about it. So I’m much more confident now, and I really enjoy it. (R2)

#20 I think you definitely have more confidence because you know that you are doing something correctly and, then you also feel that it is easier to give a presentation. (R12)

#### B2.2 Modification of attitudes and perceptions

*B2.2.1 Change of attitude towards the roles of a family physician*

Most respondents indicated that the module changed their perception of their role as family physician. Before the module they were entrenched in their understanding of their role as mainly a clinician, whilst after the module they had moved to embrace the important role the family physician has in teaching and learning.

#21 It made me just more aware of the fact that, because I am a family physician now, it will be expected of me to teach others. I have never seen it like this. I only thought I am a family physician, a better doctor and when I work in the ward, I will teach… my junior colleague … because I have more skills and knowledge. However, by doing the Modules in ‘Teaching and Learning’, I realised that it will also be my role as family physician to train nurses… or to train doctors… (R1)

This was more apparent with respondents who were appointed in family physician posts (public sector), less so for those in other type of jobs such as in a managed care environment (private sector). In exploring possible reasons for that, some indicated that being equipped with teaching tools helped them to change their role perception. One of the respondents also described how she gained insight in her role toward her patients, and how important her teaching skills are also in the doctor-patient relationship.

#22… as a student, before I did the module, I took it as an MMed student, I didn’t think about teaching as such. Yes, you interacted with students, but once you do the module, you realise that part of being a specialist is also being able to teach the subject… So it definitely influenced me positively. (R4)

*B2.2.2 Change of attitude towards teaching and learning*

The change of respondents’ attitude towards teaching that came about as a result of the module emerged as a strong theme. Some previously thought of teaching as “boring” or a duty. (cf. quote #8) Most were initially not keen on doing the module and were unwilling teachers (cf. too Quote #26). If they did do some teaching it was mainly didactic, and if opportunities for teaching came about they were not keen to pick up on them. The module facilitated a journey of self-discovery for respondents, enabling them to look at teaching from a different perspective. Some indicated that they discovered a passion for teaching that they were unaware of; whilst others indicated that the module rekindled a keen interest in teaching. For some respondents, the module resulted in a complete change of heart (cf. Quote #8).

#23 In doing this module I realised for the first time that I have a passion for training. (R3)

Furthermore, the respondents indicated that their conceptions of teaching changed from teaching-centred to learning-centred, from telling to engaging (cf. also Quote # 8).

#24 The most important thing I think was to think more of the learner than of the teacher in the context of … teaching and learning…. So, to think of what that person needs to know, rather than so much what the topic is … and how to get through to the learner and how to involve the learner. (R7)

#25 Before the teaching and training module … I just thought that I had to talk and [the students] had to listen … whereas after that I discovered that … if they present the patient …and… discuss the patient rather than me saying what we did, they would learn more because they would be actively involved … So even if they made mistakes, … it’s very important to have the students involved, that it’s not a one-way process. (R2)

These conceptions translated into behaviours as described above.

#### B2.3 Acquisition of knowledge and skills

Throughout the interviews, the respondents recalled various aspects of the module and consistently used the terminology introduced to them during the module. Together with their changed attitudes and perceptions, there was thus some indication of the acquisition of the tools with which to participate in a new, teaching-related discourse.

The respondents repeatedly mentioned various teaching skills that they not only acquired during the module but that they also used. These included presentations skills, PowerPoint skills, public speaking skills and the use of verbal and non-verbal communication skills. The use of interactive skills such as small group techniques, audience participation techniques, including participant feedback in teaching, using a range of teachings aids, and the use of tools such as the one-minute preceptor in clinical teaching were also reported widely. Respondents also emphasised how they now do careful planning of their teaching (see A1 Change in Behaviour, above).

#26 I did it because I had to. But I think I was quite surprised at how much I learnt, and appreciated it. Well, I think some of it just confirmed what I already knew, and so reinforced that. But I actually particularly enjoyed the project, the assignment we had to do, because I still remember clearly what I did and I actually used it. (R5)

#27 I think what sort of stands out the most for me is that it kind of put things in order. But if you think about it in terms of the way the module said plan your work and think about how you are going to address the learning needs of your audience or how would you evaluate that this was a successful talk or presentation, in that sense definitely. It kind of put everything into a structured way and made it easier for you to handle a teaching task. (R4)

## Discussion

Our aim with this exploratory project was to investigate the influence that a module on teaching and learning has had on family physician graduates. We have documented a suite of self-reported changes in teaching behaviours that have evidently resulted from participating in the module. Although this study was not designed as a systematic evaluation of the achievement of the outcomes of the module, our findings map strongly to the module’s stated outcomes (Box 1), except for principles of assessment (which have subsequently been dropped from the module). The inclusion of graduates as researchers added richness to the evaluation process based on their experience and insider knowledge of the module.

We have also documented benefits accruing to the professional and personal lives of respondents and, to some extent, those around them. Some graduates involved and supported other reluctant teachers in teaching activities and helped them gain skills and confidence. These changes have persisted a few years after completion of the module for some respondents, adding to limited evidence of longer term changes of educational interventions [10]. Graduates have also incorporated what they learned into quality improvement activities.

These results add to the evidence that exposing family medicine residents to teaching knowledge and skills improves their self-confidence and changes their self-reported teaching behaviours. Although this improvement is well described in the international literature [5], little if any such work has been done on the African continent, where such courses are rare. In this sense our work thus anchors the relevance of the global evidence in our context.

While we are unable to draw causal inferences between the various types of changes we have demonstrated, the changes are such that, together, they are likely to predispose the residents to improved teaching behaviours. The attitudes and attributes, knowledge, and skills of competent teachers were recently described [11]. Amongst others, demonstrating passion as a teacher, acknowledging that the goal of effective teaching is directed at effective learning and understanding, employing basic pedagogic principles, demonstrating basic skill for effective lecturing and facilitating small group learning, identifying learners’ needs, and being a reflective mindful teacher were listed. These are all qualities that emerged from our results. In particular, we have documented a shift in attitudes towards, and conceptions of, teaching. Whether this is of benefit to the students these graduates teach is cause for further study, but there is reason to believe that this shift may well benefit students [12].

Perhaps the most interesting of our findings is that the TLFM module broadened the trainees’ conception of their role as family physician from primarily that of clinician to also include being a teacher. The first two weeks of the module are spent on clarifying the role of the family physician as a teacher. Our results support previous findings that residents who had participated in a residents-as-teachers curriculum were more likely to enjoy teaching and want to participate in teaching in the future than their peers who had not participated in the curriculum [13]. There is evidence that a focused teaching intervention improving teaching skills of residents leads to an increased interest in pursuing faculty positions [13, 14]. This has important implications not only for the development of family medicine but also for recruitment of future medical teachers in African medical schools [15].

As a substantial part of the module was completed via an online platform with extensive asynchronous discussions, we were curious to understand whether community of practice theory could apply to our results. Communities of practice are described as “groups of people who share a concern, a set of problems, or a passion for something they do, and who deepen their knowledge and expertise in this area by interacting on a regular basis” [16]. Online mediated learning offers opportunities for student-student and student-tutor interaction and discussion while geographically separated. Both these activities are important aspects of learning as a social activity. Embedding structured, asynchronous online discussions with peers and tutors in the online learning environment can create a community of practice in support of the social nature of learning, given the dispersed and often isolated by a lack of face-to-face interaction [17]. Although the influence of the community of practice on the students and their learning was not the focus of this research interviewees did refer to the value of the feedback they received during the module. This should make for interesting future research.

Although the module was not explicitly designed with the theory of learning transfer [18, 19] in mind, clearly, aspects of the design have enabled participants to apply and even adapt what they have learned in their respective professional contexts, even under adverse conditions. One factor may be the incremental experience participants acquired developing and trying out their knowledge and skills as part of the module. For some, the micro-teaching they undertake at the end of the contact day is their first teaching presentation, often their first presentation using PowerPoint too. Subsequently, participants are accompanied in the process of developing and executing a more extensive teaching activity. The module content also includes evaluating one’s teaching activities and adjusting accordingly. These elements of the design of the programme seem to inculcate a sense of self-confidence, agency even, among respondents that subsequently stands them in good stead.

Our results also highlight the interplay between self-confidence and context. The opportunity to initiate or become involved in teaching activities varied greatly. The application of what was learned was evidently a function of what we call opportunity pressure, the pressure to have to undertake teaching, and self-confidence: application = [opportunity pressure] × [self-confidence]. For some, the opportunity pressure was very great and had to be acceded to irrespective of self-confidence. For others, their self-confidence was such that they grasped the slightest opportunity to teach or even created opportunities to teach. This was something many admitted they would not have done prior to completing the module. The very fact of subsequently getting involved in teaching allowed graduates to hone their teaching skills and further built their self-confidence.

Initiating or getting involved in teaching occurred whether or not graduates necessarily had the positional power to do so. Nonetheless, being appointed in a position where teaching was a clear expectation of the job certainly, made it easier. Some respondents were perhaps in a better position to make decisions on and start teaching initiatives than others who were in lower ranking positions. In conjunction with having the opportunity to teach, however, having the capacity also played a role i.e. not being so overburdened by clinical service that there is no time or “head space” to contemplate teaching better or developing new teaching activities. The biggest barrier to effective teaching was identified as time available, a factor that has previously been highlighted [20, 21]. This emphasises again the importance of protected time for teaching in the service environment and therefore lends support to the recommendation that providing protected time for learning should be part of facility managers’ performance evaluation [20]. If clinicians’ performance evaluation only takes heed of such factors as number of patients seen, this is likely to remain a serious impediment to ensuring effective teaching in clinical facilities outside of teaching hospitals. This will also serve as a barrier to extending clinical training platforms to expand capacity to educate health care professionals.

Although these results clearly indicate the respondents’ change in knowledge, skills and attitudes as a result of the module, a clear limitation of this study is that the results remain the perceptions of respondents of their behaviour change and the effectiveness of their teaching. We are not able to comment on the quality of the behaviours reported or whether the learners taught by these individuals benefited. Future research should include exploring whether participants’ future learners receive more thoughtful and more effective teaching than do those taught by “occasional teachers” who have not completed this or a similar, module. This could include expert or peer observation of teaching. The contexts of our 13 participants may not reflect those of family physicians elsewhere in South Africa and Africa in terms of factors like available teaching resources, resourcing of the clinical environment and support of managers. However, the purpose of this study was to explore the effects of this module on graduates of this course. Although respondent numbers were limited, respondents worked in a variety of contexts and data saturation was achieved during analysis. The focus on graduate perception of the programme is also a limitation, as individual action and behaviour were influenced by the participants’ perception of how the programme changed them.

Given that our results suggest that a TLFM training course can positively influence both teaching by residents and their understanding of their role as a leader in their chosen profession, the question arises whether a module such as this should not be standard fare in postgraduate medical education programmes, especially in resource-constrained environments. Much clinical teaching of undergraduate medical students is undertaken – by default or by design – by residents. These residents are ill-equipped to undertake this task. Findings suggest that they are possibly more likely to consider and enjoy a career involving teaching if they have even a basic preparation for their teaching role [13]. We would argue that incorporating such a module into all postgraduate medical programmes would augur well for both the quality of undergraduate teaching and the future of the academic profession. Equally inasmuch as we would be equipping current teachers and future leaders to tackle their teaching role more effectively, we would also be instilling in them an understanding of the multifaceted nature of being a leader in the field, a role that includes teaching alongside other facets like advanced clinical care and research.

## Conclusions

We believe we have succeeded in achieving the aim of this module, namely to prepare family physicians to be effective teachers of family medicine in her/his own setting. In fact, although focussed on teaching and learning, this module appears to develop trainees’ abilities as capacity builders. Whether this ultimately translates into improved health care is an ideal that remains to be explored.

## Authors’ information

FC and KvP were practising as Family Physician specialists in the Cape Winelands District of the Western Cape Department of Health, South Africa, as well as preceptors of undergraduate and postgraduate students on Stellenbosch University’s Rural Clinical School at the time of the study. They were invited to join the research group as two graduates who had recently completed the TLFM module. KvP joined the Division of Family Medicine and Primary Care, Stellenbosch University, as Senior Lecturer and Research Project Coordinator in April 2014. MRdV, FJC and NH designed, implemented and tutored on the TLFM module since its inception. MRdV is a family physician, holds a PhD and is Deputy Dean: Education, Faculty of Medicine and Health Sciences, Stellenbosch University. FJC holds an MPhil in higher education, a PhD in health professions education (and a medical degree from his ‘first life’) and was the Deputy Director at the Stellenbosch University Centre for Teaching and Learning at the time of the study. He is currently Associate Professor, Education Development Unit, Faculty of Health Sciences, University of Cape Town. NH holds an MPhil in higher education and is currently a senior advisor in higher education at the University’s Centre for Teaching and Learning. She has been the driving force behind the educational development initiatives for newly appointed academics and is currently completing a PhD in the field. MvH was a part time research assistant in the Dean’s Division, Faculty of Medicine and Health Sciences, Stellenbosch University at the time of the study and is currently a part-time lecturer with the Language Centre, Stellenbosch University.

## Abbreviations

TLFM: Teaching and learning in family medicine
TB: Tuberculosis
MMed: Master of medicine.
